# Emerging structural and pathological analyses on the erectile organ, corpus cavernous containing sinusoids

**DOI:** 10.1002/rmb2.12539

**Published:** 2023-09-01

**Authors:** Daiki Hashimoto, Kota Fujimoto, Sang Woon Kim, Yong Seung Lee, Masanori Nakata, Kentaro Suzuki, Yoshitaka Wada, Shinichi Asamura, Gen Yamada

**Affiliations:** ^1^ Department of Developmental Genetics, Institute of Advanced Medicine Wakayama Medical University Wakayama Japan; ^2^ Department of Physiology and Regenerative Medicine, Faculty of Medicine Kindai University Osaka Japan; ^3^ Department of Plastic and Reconstructive Surgery, Graduate School of Medicine Wakayama Medical University Wakayama Japan; ^4^ Department of Urology, Urological Science Institute Yonsei University College of Medicine Seoul South Korea; ^5^ Department of Physiology, Faculty of Medicine Wakayama Medical University Wakayama Japan; ^6^ Faculty of Life and Environmental Sciences University of Yamanashi Yamanashi Japan

**Keywords:** corpus cavernosum, erectile dysfunction, erection, penis, sinusoids

## Abstract

**Background:**

The corpus cavernosum (CC) containing sinusoids plays fundamental roles for erection. Analysis of pathological changes in the erectile system is studied by recent experimental systems. Various in vitro models utilizing genital mesenchymal‐derived cells and explant culture systems are summarized.

**Methods:**

3D reconstruction of section images of murine CC was created. Ectopic chondrogenesis in aged mouse CC was shown by a gene expression study revealing the prominent expression of Sox9. Various experimental strategies utilizing mesenchyme‐derived primary cells and tissue explants are introduced.

**Main Findings:**

Possible roles of Sox9 in chondrogenesis and its regulation by several signals are suggested. The unique character of genital mesenchyme is shown by various analyses of external genitalia (ExG) derived cells and explant cultures. Such strategies are also applied to the analysis of erectile contraction/relaxation responses to many signals and aging process.

**Conclusion:**

Erectile dysfunction (ED) is one of the essential topics for the modern aged society. More comprehensive studies are necessary to reveal the nature of the erectile system by combining multiple cell culture strategies.

## 1 INTRODUCTION

External genitalia (ExG) is the essential reproductive organ. Corpus cavernosum (CC) in such an organ plays essential roles for erection.^1^ Sinusoids, the microvascular complex inside the CC, consist of endothelium and smooth muscle cells, which are filled with blood and dynamically expand during erection.^2^ It has been suggested that cellular and histological changes result in erectile dysfunction (ED) which is getting frequent in the current aging society.^3^, ^4^

To understand the mechanism of dynamic erectile functions, various experimental models have been utilized.^5^, ^6^, ^7^ They contribute to understanding of the mechanisms for erection such as the signal cascade of nitric oxide synthase (NOS)‐cGMP axis.^8^ Generally, recent urological research showed significant advances in the research field for NO signal and local neuronal regulation.^9^, ^10^ There are growing concerns about pathological mechanisms of ED and the effect of aging on the erectile structure including fibrosis and aberrant chondrogenesis.^11^, ^12^ However, analyses of such pathological changes inside the erectile system, the sinusoids has rarely been studied through only a few histological studies.^13^, ^14^ To analyze such mechanistic details, new experimental systems on the character of genital mesenchyme are necessary. In the current review, various in vitro culture models utilizing mesenchymal‐derived cells and tissue explant culture systems are summarized. Mesenchyme‐derived primary cultured cells and a few cell lines are first introduced. In addition, a novel in vitro explant system reproducing the erectile contraction/relaxation process is summarized.^15^, ^16^ The system should be utilized to perform various analyses of erectile pathogenic processes.

## 2 THE STRUCTURE OF ERECTILE TISSUE IN THE MALE REPRODUCTIVE ORGAN

**FIGURE 1:**
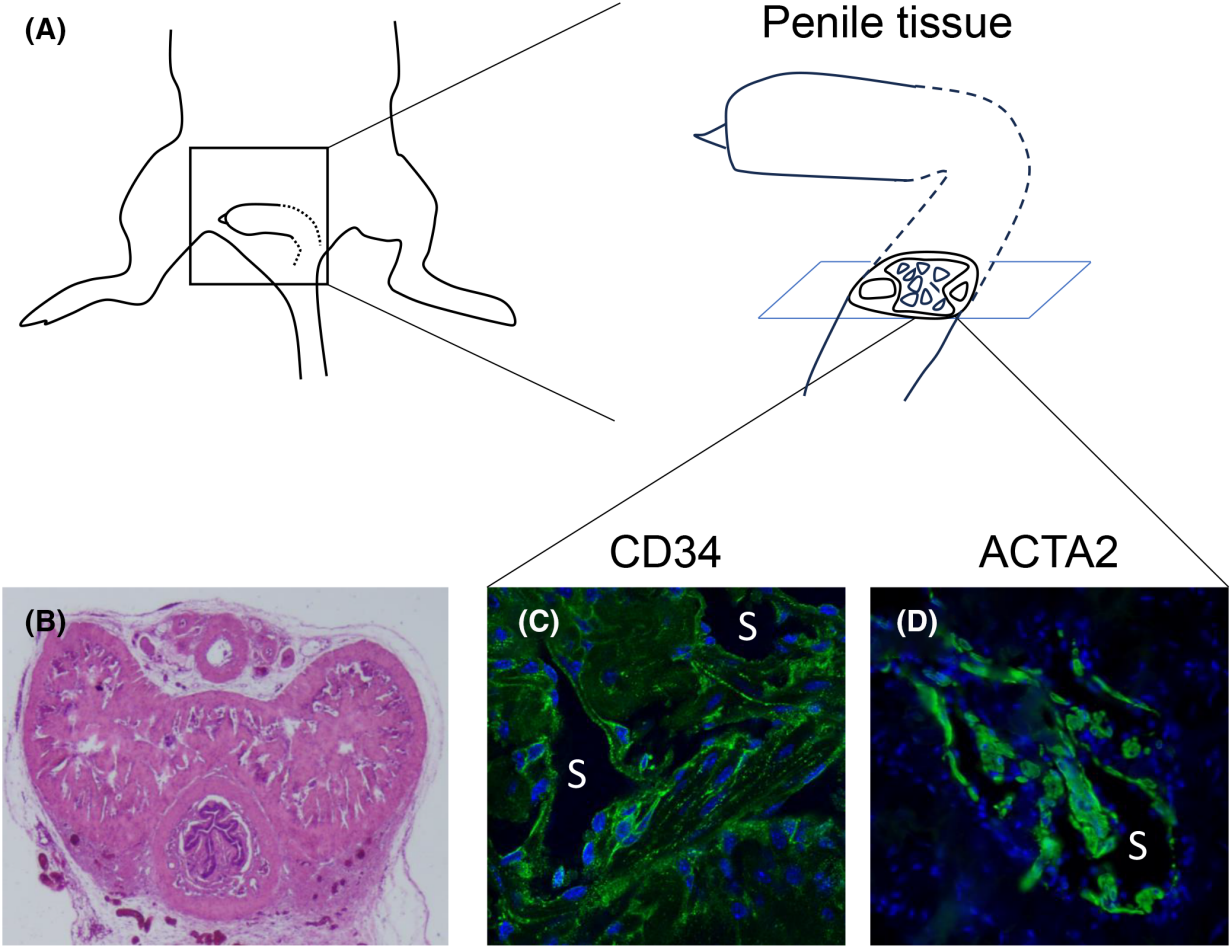
The structure of mouse corpus cavernosum (CC) and its immunostaining analyses. (A) An illustration showing the location and structure of adult mouse penis. (B) The cross‐section of hematoxylin–eosin (H.E.) staining of CC. (C, D) The images of immunofluorescence staining for the CD34‐positive endothelial cells and ACTA2‐ (alpha‐smooth muscle Actin) positive smooth muscle cells. S, Sinusoidal spaces.

ExG contains three corporal bodies, corpus cavernosum (CC), corpus cavernosum glandis (CCG), and corpus cavernosum urethrae (CCU; Figure 1A).^17^ In the case of mouse CC, it mostly locates inside the pelvic region,^18^ unlike the human cases in which the majority of penile tissues locate outside of the perineal region. Inside the erectile tissue, blood flow from the deep artery (DA) reaches to sinusoids via the helicine artery and subsequently to the venous system.^19^, ^20^ Each sinusoid contains endothelial cells, smooth muscle cells and blood flow into the sinusoids leads to their expansion which is necessary for erection. Subsequently to the erection, the sinusoidal blood flows through tunica albuginea to venous system and to the dorsal vein which leads to the penile flaccid status.^21^

A scheme is shown for the location and structure of the adult mouse penis (Figure 1A). The mouse external penile region corresponds to the glans in flaccid conditions and the location of major CC is shown, which contains prominent sinusoids adjacent to the dorsal vein and artery (Figure 1B). Various types of cells in the CC are demonstrated including CD31‐positive (also positive for CD34) endothelial cells, ACTA2‐ (alpha‐smooth muscle actin) positive smooth muscle cells (Figure 1C,D), and NG2‐positive pericytes located adjacent to tunica albuginea.^22^

**FIGURE 2:**
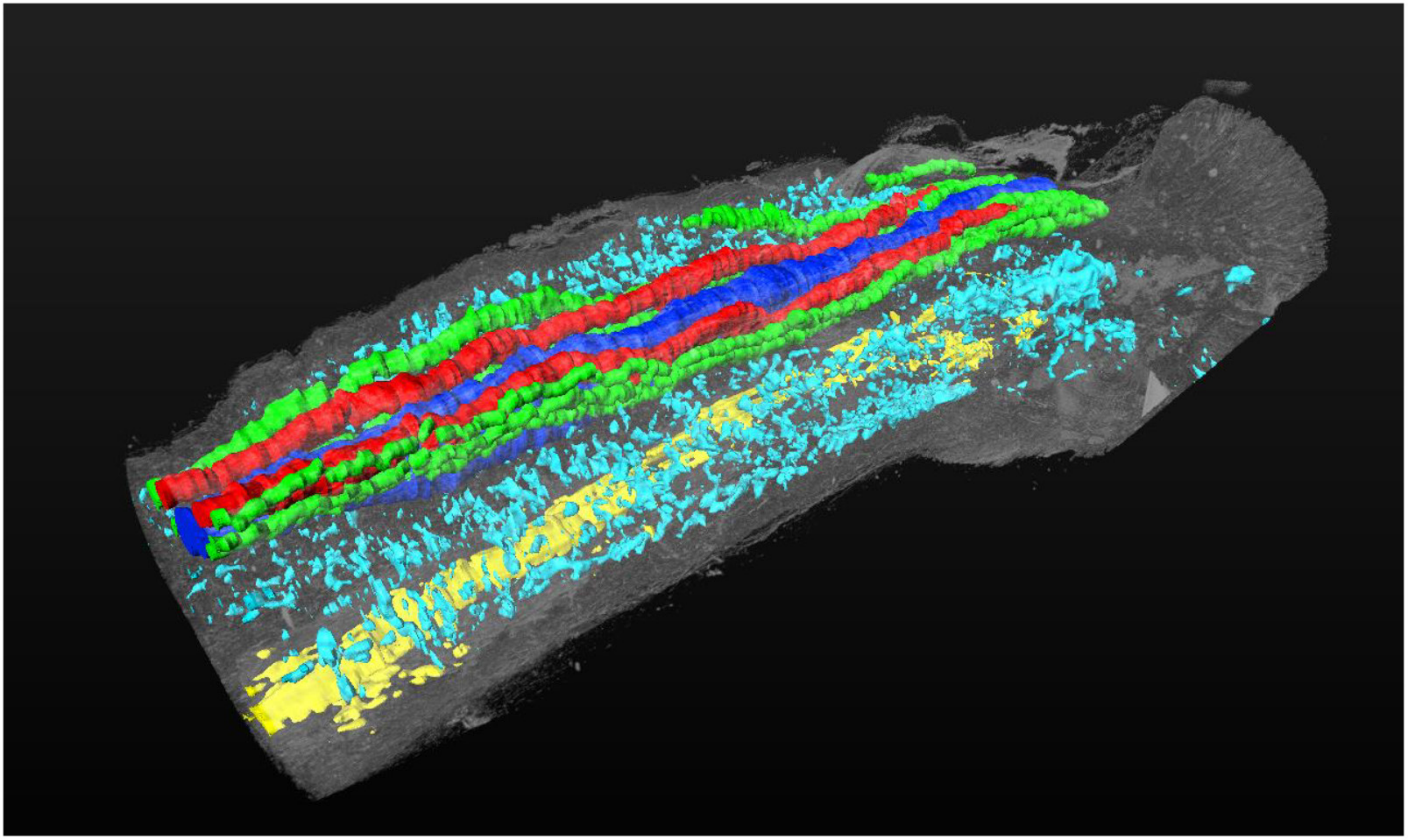
Three‐dimensional (3D) reconstruction of normal mouse penis. The regions corresponding to proximal and distal end of external genitalia are right and left side, respectively. The cyan color regions indicate sinusoidal spaces. The red color regions indicate dorsal artery. The blue color regions indicate dorsal vein. The green color regions indicate nerve bundle. The yellow color regions indicate urethra.

To understand the complex structure of the erectile tissue, 3D reconstruction of murine CC with sinusoids was created by incorporating histologically stained sections.^23^ A significant number of sinusoids is observed located in the outer regions of CC adjacent to the tunica albuginea. The identified sinusoidal structures show various sizes and structures shown by such reconstructed 3D images (Figure 2, dorsal diagonal view of CC containing sinusoids). Dorsal midline structure contains dorsal artery and veins. In the bilateral region, well‐developed sinusoidal structures are observed which show various sizes. In addition to sinusoids, mouse CC contains the prominently developed collagen‐rich trabecular structure concentrated in the middle region (island like central area) which extends radially toward the outer region.^5^, ^16^, ^23^, ^24^ Thus, central CC region may offer structural and mechanical supports for the radial expansion by erection. The prominently developed sinusoids located adjacent to the outer of CC may suggest the essential roles of “outer” zone for erection during contraction/relaxation.^23^ Such collagen‐rich supporting structures for sinusoids are also studied in pathological processes. In addition to the character of outer sinusoidal region of CC, the region close to the tunica albuginea should be also noted. It has been known that tunica region is considered responding to mechanical stress due to erection and contraction.^25^, ^26^ Hence, the region is prominent for containing collagen and elastin.^26^, ^27^ Organic ED has been also suggested in relation with the reduction of veno‐occlusive functions^28^, ^29^ and certain type immature cells including slow mitotic cells have been detected adjacent to tunica region.^30^, ^31^ Further studies are necessary to investigate cell populations including the sub‐tunica region.

The structural information of CC can also be linked with current therapeutic strategies. The treatment of ED was dramatically improved by the administration of phosphodiesterase 5 (PDE5) related inhibitors such as sildenafil (Viagra).^32^, ^33^ However, there are still clinical cases showing resistance to such treatment, and direct drug injection into the penis has been often performed such as prostaglandin E to exert its function inside the CC.^34^, ^35^, ^36^ However, the current research situation lacks injected drug‐distribution analysis related with sinusoids. Hence, injected drug in the sinusoidal structure should be analyzed and its dispersion from the injected site to other parts of the sinusoids inside the CC requires more attentions.^37^

## 3 ERECTILE PATHOGENIC PROCESSES, MESENCHYMAL CHONDROGENESIS

Erectile tissue containing CC shows unique tissue character as a copulatory organ. Because it needs to function as an insertional organ for ejaculation, its unique development and pathogenesis are known.^38^, ^39^, ^40^, ^41^ The copulatory organ has been described for significant structural and functional diversification among species.^42^, ^43^, ^44^ Mouse erectile system contains the penile bone (baculum) which develops gradually around puberty.^45^, ^46^ Unlike human penises, many mammalian species develop baculums which are solid for copulatory insertion and the coexistence of bone and chondrogenic structure inside the erectile tissues has been known.^43^, ^47^ Thus, CC offers an intriguing research object from evolutional and comparative biological viewpoints.^42^

**FIGURE 3:**
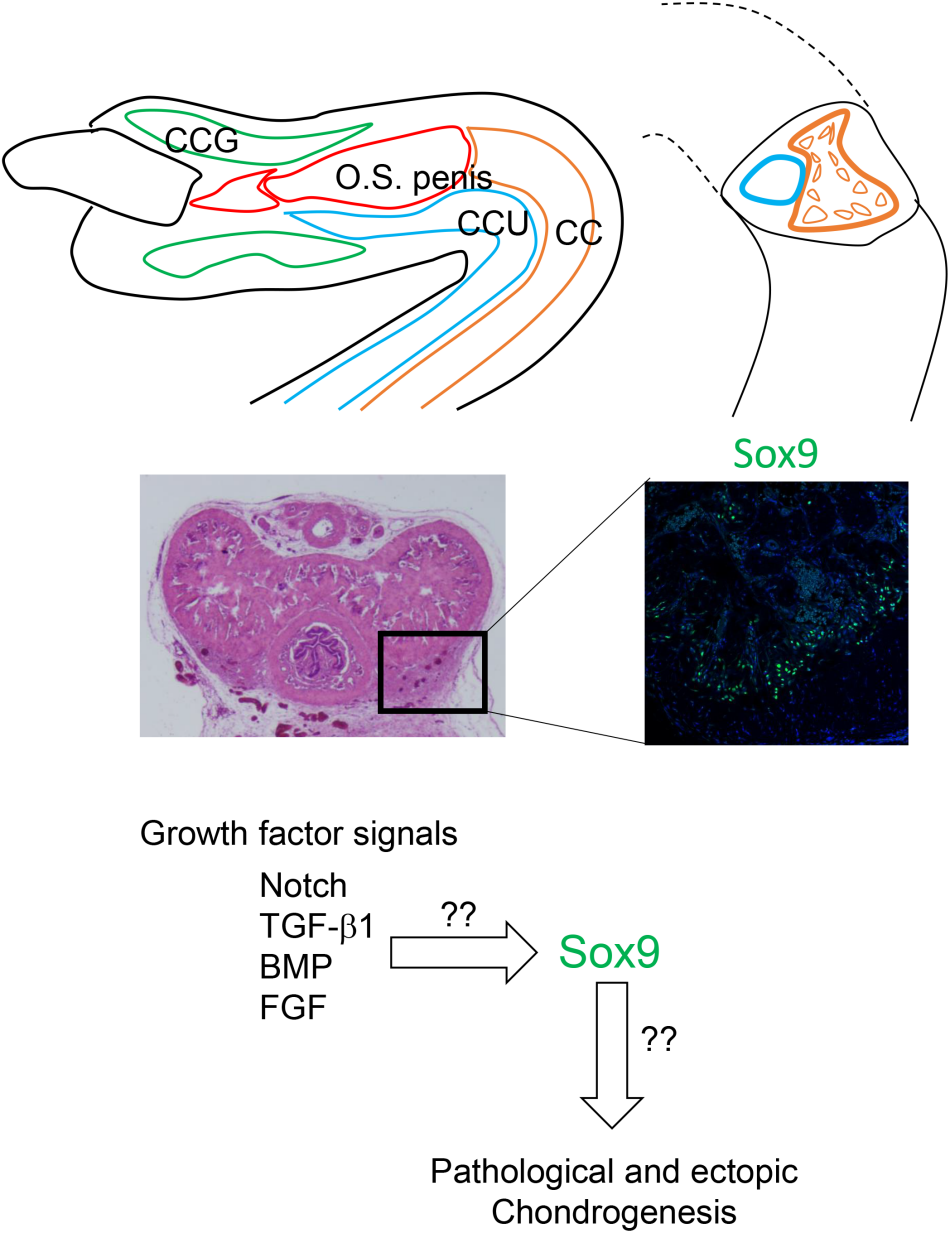
The structure of mouse penis and detection of ectopic SOX9 expression in the aged mouse CC. Ectopic SOX9 expression in the aged (14 months old) mouse CC (green signals in the lower right side). Black square region indicates part of the outer region of CC (including tunica albuginea) in the aged mouse CC. The schema shows possible growth factor signaling pathways including Notch which may lead to the augmented Sox9 expression and chondrogenesis.

Ectopic chondrogenesis in aged mouse CC was studied histologically and by gene expression study (Figure 3).^23^ Sox9 is one of the Sox family genes, a transcription factor with high‐mobility‐group (HMG) domain. Of note is its prominent expression in such ectopic chondrogenic region.^23^ Maintenance and homeostatic regulation are supported by cellular behaviors also for the cases of microvascular organs, sinusoids^48^, ^49^ and the proper differentiation of mesenchymal cells should be achieved for such maintenance.^50^ Ectopic chondrogenic cells detected in aged mouse model might reflect the genital mesenchymal cell characters “allowing” the development of penile bone in mice. Species differences have not been enough considered for the penile pathological processes and the existence/absence of penile bones requires more mesenchymal cell specification studies.

The ectopic chondrogenesis in human penile tissue is rarely reported. As for fibrosis, the pathogenesis of Peyronie's disease is reported.^51^ Peyronie's disease is also correlated with ED.

In the case of vascular calcification, myocardium calcification leads to reduction of its contractility.^52^ In addition to such pathogenesis, atherosclerotic aortic calcification also reduces contractile maker gene expression and its elastance.^53^, ^54^ Ectopic calcification has been often described during aging and diabetes^55^, ^56^, ^57^ and there are growing concerns about certain similarities of embryonic bone/chondrogenesis compared with pathogenic calcification. Recently, analyses of the calcification process based on altered cellular differentiation of mesenchymal cells, endothelial cells, and smooth muscle cells have been investigated.^58^, ^59^, ^60^, ^61^ Abnormal cellular differentiation has been described from mesenchymal stem cells (MSCs) toward such chondrogenic cells.^62^ Other types of calcifications have been also described as a sort of trans‐differentiation in which mesenchymal or endothelial cells have differentiated to chondrogenic cell linages.^63^ Further studies are necessary to compare the relationship of calcification and contraction/relaxation in vascular system and sinusoids.

Related with cardiovascular and penile abnormalities, it has been suggested that the symptom of ED is one of the factors “predicting” subsequent cardiovascular dysfunctions.^64^ Such cardiovascular dysfunction includes coronary heart diseases (CHD), stroke and severe mortality cases.^65^ Because severe form of CHD should be treated at early stages, the phenotypes of ED should be considered not only as a reproductive disease but also as early symptoms of cardiovascular diseases. Functional and pathological examination of penile‐vascular system should be thus further considered.

## 4 REGULATORS FOR ECTOPIC CHONDROGENESIS AND AGING; SOX9 AND GROWTH FACTOR SIGNALS

To reveal the mechanisms of erectile pathogenesis, investigation of several regulators is necessary. As for chondrogenesis, regulation of its key regulator, *Sox9* gene, has been described in the developmental context.^66^ During sexual differentiation, Sox9 is one of the key regulators for testicular differentiation^66^, ^67^ and involvement of its regulators has been suggested.^68^ Its regulation has been also described for the case of growth factor signaling^69^, ^70^ and they include regulatory factors such as Shh (Sonic hedgehog) and Wnt‐related signals.^71^, ^72^, ^73^, ^74^ In case of vascular system, Wnt signal has been suggested for promoting pericyte differentiation to chondrocytes but Sox9 was basically demonstrated as a marker for such differentiation.^75^

Of note is the prominent embryonic Sox9 expression in both male and female presumptive CC inside the GT.^76^, ^77^ Furthermore, conditional knock out mouse studies for β‐catenin, which is the critical canonical Wnt signaling regulator, identified potential role of Sox9 for regulating such embryonic sex‐independent mesenchymal cell proliferation.^76^ Therefore, regulation of Sox9 is also critical in early staged mesenchyme as well as late staged male type CC formation.^76^ Previous studies suggested the androgenic CC formation with sinusoids occurs in pubertal period.^76^ Altogether, investigation of Sox9 and Wnt signal should be further studied in both stages, that is, sex‐independent and ‐dependent CC formation and pathogenesis.

For the analysis of mesenchymal cell responses to Wnt signals, the dicorrelation of Wnt signaling and Sox9 has not been suggested for bone development and previous works suggested its involvement for bone development and absorption.^78^, ^79^, ^80^ Up to now, several types of in vitro studies for chondrogenic or osteogenic processes have been performed utilizing cell lines.^81^, ^82^ In mouse experimental systems, ATDC5 cells derived from teratocarcinoma cells have been utilized and growth factor response analysis including Bone morphogenic protein (BMP) signaling was performed.^83^, ^84^ Further applications of such system including the establishment of chondrogenic cell line from mesenchymal cells of ExG remain to be performed.

As for other signals of vessel calcification processes, involvement of Notch signaling has been suggested for the case of vascular smooth muscles.^85^, ^86^ Notch gene family consists of regulatory factors which are involved for multiple cellular events including chondrogenesis and pathogenic proceses.^87^, ^88^, ^89^ In order to examine the status of regulatory signals for such ectopic chondrogenesis, the expression of Notch‐related regulators was examined.^23^ In vascular smooth muscle progenitor cells, sustained Notch/Jag1 signaling suppresses the expression of chondrogenic genes including Sox9.^90^ It has been also reported that RBPJ/Notch intracellular domain (NICD) complex represses Sox9 transcription.^91^ When such cascades are disrupted, detection of ectopic chondrocytes and abnormal vascular calcification has been reported.^92^ The correlation between Notch signaling and chondrogenesis is also reported for animal models and cell line experiments.^93^ How similar mechanisms exist for the SOX9 gene regulation in erectile tissues awaits further investigations. Possible similarity and diversification of normal and pathogenic mesenchymal chondrogenic programs of erectile system should be studied.

## 5 IN VITRO CULTURE SYSTEMS TO ANALYZE GENITAL MESENCHYMAL CHARACTERS

**FIGURE 4:**
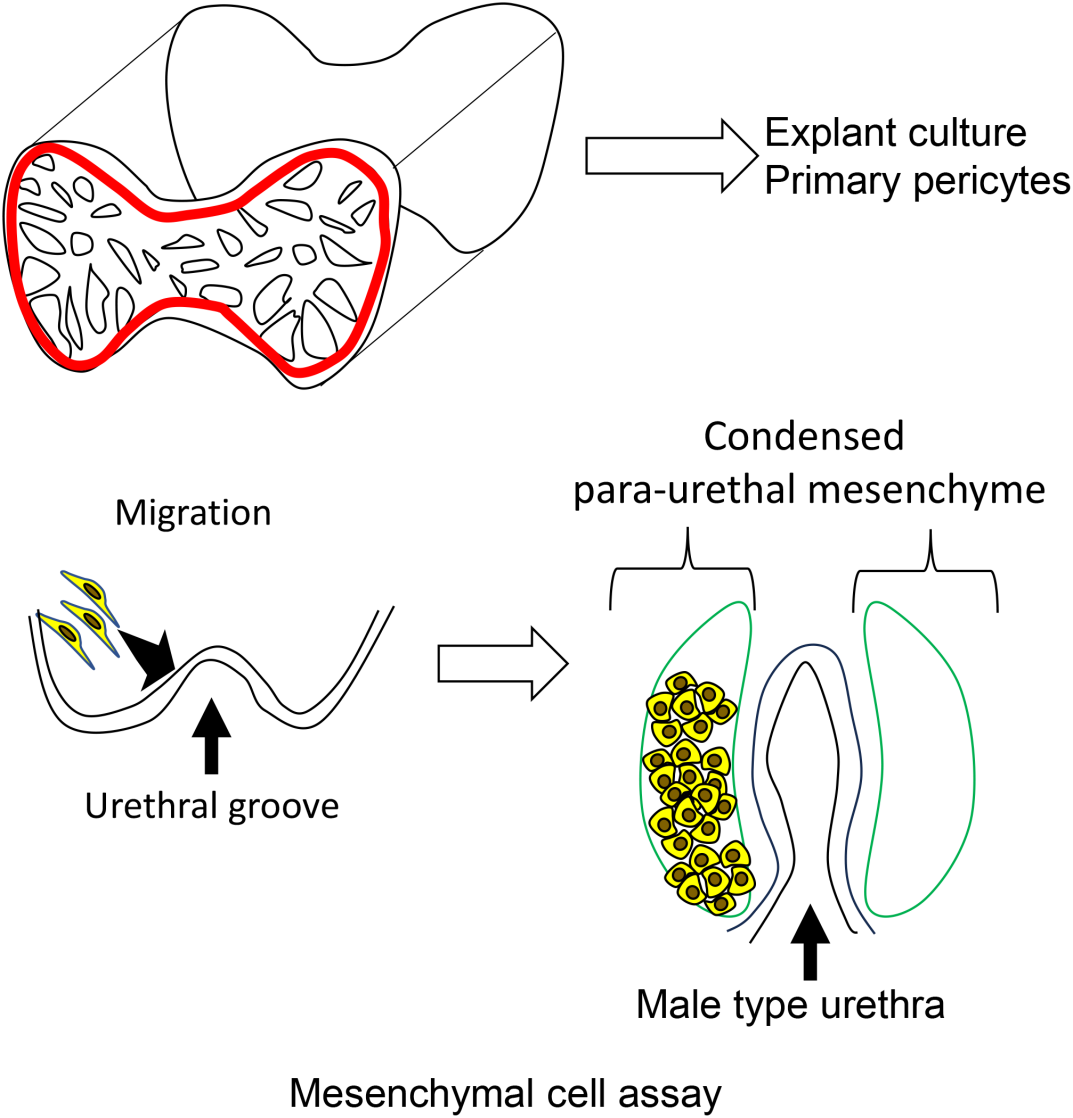
The schema showing the location and utility of explants and primary cells from external genitalia (ExG). Explants culture from CC can be utilized for physiological and pathogenic analyses (upper row). Mesenchymal primary cells can be applied for cell behavior analysis for para‐urethral region and for mesenchyme‐derived pericytes (upper and lower row).

The unique character of genital mesenchyme is shown by various analyses of ExG utilizing mesenchyme‐derived cells.^94^, ^95^, ^96^ Cellular characters of mesenchyme should be considered for both CC and other regions of ExG, the para‐urethral mesenchyme (Figure 4). Many conditional knock‐out mice analyses which specifically modulate epithelial (such as the case of Shh) and mesenchymal (such as the case of MafB, BMP) signals have identified that such epithelial or mesenchymal regulators are essential for embryonic urethral development. Such important cellular characters include cell migration, adhesion/aggregation as part of the epithelial‐mesenchymal interaction (EMI). Establishment of the stable mesenchymal cell lines derived from ExG has not been performed. Primary cell lines from in vivo urogenital tissues have been utilized and cells from embryonic genital mesenchyme behave similar like mesenchyme around the embryonic urethra. Such cells migrate prominently toward the midline urethral plates by androgen to develop the condensed mesenchymal mass^94^, ^96^ and the migration/condensation is essential for male type urethral development^97^ (Figure 4). The migration/condensation is tightly linked with the formation of urethral region and other parts of ExG, such as CC. MafB, AP1 superfamily transcription factor, is expressed in para‐urethral mesenchyme and its regulation of cellular proteins including actomyosin is currently under investigation.^94^, ^98^, ^99^ In the male type urethral formation, growth factor signals including canonical Wnt signal may interact with other signal cascades including AP1. Hence, different growth factor signals act coordinately on various genital mesenchymal cells regulating their migration and primary cells should be thus more studied.

Mesenchymal migratory behaviors and adhesive characters can be analyzed also for erectile tissues because mesenchymal cell condensation has been detected during embryonic CC development.^76^ Along with such condensation, proper input of canonical Wnt signaling has been shown.^76^ In addition, local conversion of testosterone to dihydrotestosterone (DHT) by SRD5A enzyme has been also suggested in the region.^38^ Possible actions of local androgen (DHT) and other signals regulating CC and mesenchymal development still require analyses. Related with the cell migration for CC, the role of androgen induced migration of pericytes has been described.^22^ Pericytes are one of the unique and important mesenchymal cell lineages which are regulated by concerted action of growth factor signals including PDGF (Platelet‐Derived Growth Factor) and VEGF (vascular endothelial growth factor).^100^ Its association after the migration with the blood vessel endothelium is the key event regulating the nature of such vessels. How such pericytes forming the vasculature rich sinusoidal structure remains to be examined. In the case of CC, sort of loose types of association may be responsible for the flexible‐expandable nature of sinusoids.^22^ About the origin/function of pericytes, pericyte distribution in sub‐tunica region is reported.^22^ Furthermore, they can migrate out from the in vivo‐derived explants and their cellular behavior responding to androgen should be further examined. Another important topic of cell migration for CC is the therapeutic administration of various MSCs into penis for ED treatment. Cell therapy is one of the growing strategies to treat the damaged tissues. MSCs including hematopoietic MSCs have been administrated and how administrated MSCs migrate in the CC and sinusoid remains to be analyzed.^101^, ^102^

The cellular phenomena of mesenchymal migration/condensation are tightly linked with cell–cell interaction in the EMI. In the case of genital mesenchyme for urethral formation, emerging events after the migration should be noted. How the basement membrane (BM) participates in EMI is a growing research field. The disappearance of the urethral plate epithelia is associated with the breakdown of the BM between urethral plate and the adjacent bilateral mesenchyme of urethra.^94^, ^96^ The changes in the BM could be mediated partly by the epithelia and the epithelial Shh can induce the deposition of laminin in the BM. In fact, loss of Hh signaling results in defective myotome in both mice and zebrafish models.^103^ In the zebrafish myotome, epithelial Shh‐induced laminin deposition inhibited BMP signaling in the mesoderm leading to myotomal cell fate specification. Thus, Hh from the urethral epithelia might participate in paracrine signaling via the BM for the urethral formation. Hh signal has been also suggested as expressed in CC and its involvement for diabetes mediated ED has been indicated.^104^, ^105^

Another application of primary cells and explants is co‐culture type experiments. It has been suggested that co‐culture of penile pericytes and endothelial cells is effective for the analysis of CC pathogenesis.^22^ It has been also suggested that the interaction of para‐urethral mesenchyme versus nearby urethral plate epithelial cells is essential and such interaction may be analyzed by co‐culture type experiments (Figure 4). Altogether, experiments with primary cells and explants and co‐culture experiments should be further applied.

## 6 CC DERIVED PRIMARY EXPLANT CULTURE SYSTEM TO ANALYZE ERECTILE CONTRACTION/RELAXATION FUNCTIONS

Primary cells and explants can be utilized for additional research purposes. Tissue explant systems have been analyzed in vitro for reproductive functional researches.^15^, ^23^ Recently, development of the explant culture system visualizing the dynamic processes of sinusoidal expansion, contraction/relaxation, of the mouse CC was performed^15^, ^16^, ^106^ (Figure 4). Such explant system can visualize contraction/relaxation behavior and the effects of external factors for erectile functions in vitro. Administration of prostaglandin E has been often utilized to treat ED patients which show resistance for PDE5 inhibitor treatment and its effectiveness has been shown by such explant culture system.^16^ One of the advantages of such system is its capability to repeat contraction/relaxation processes in vitro. For vascular smooth muscle research, induction of contraction for a few times to examine their contractile responses was performed. To examine the effects of repeated contraction/relaxation of CC tissues, PE (phenylephrine) induced contraction and SNP (sodium nitroprusside)‐induced relaxation was performed. Intriguingly, CC derived explants still show contraction/relaxation after such repeated stimulations, and real‐time PCR was performed to examine the stress status. Expression of oxidative stress marker genes, *Hif1a*, *Gpx1*, and *Sod1*, was significantly increased. Ras homolog family member A (*RhoA*), Rho associated protein kinase 1 (*Rock1*) and Rho‐associated protein kinase 2 (*Rock2*), genes have been related with contraction and their expression was also upregulated.^15^ One of the stresses of vascular systems includes Rho/ROCK pathway and oxidative stresses. Further researches are necessary to study the patho‐physiology of contraction/relaxation for erectile system.

## 7 CONCLUSION

Erectile system is “a sensitive system” for androgen and external factors including various mechanical stresses. In the current review, various experimental strategies utilizing mesenchyme‐derived primary cells and explants are introduced. Such strategies should correspond to genital mesenchymal characters performing erectile responses and to various signals and aging. Further studies are necessary to reveal the nature of erectile system by combining multiple strategies.

## CONFLICT OF INTEREST STATEMENT

The authors declare no conflict of interest.

## ETHICS STATEMENT

All procedures and protocols were approved by the committee on animal research at Wakayama Medical University, Wakayama, Japan (approval number: 995).

## INFORMED CONSENT

This work does not contain human subjects.
